# Sustainable Protein‐Based Binder for Lithium‐Sulfur Cathodes Processed by a Solvent‐Free Dry‐Coating Method

**DOI:** 10.1002/cssc.202201320

**Published:** 2022-10-20

**Authors:** Florian Schmidt, Sebastian Kirchhoff, Karin Jägle, Ankita De, Sebastian Ehrling, Paul Härtel, Susanne Dörfler, Thomas Abendroth, Benjamin Schumm, Holger Althues, Stefan Kaskel

**Affiliations:** ^1^ Inorganic Chemistry I Technical University Dresden Bergstraße 66 01069 Dresden Germany; ^2^ Chemical Surface and Battery Technology Fraunhofer Institute for Material and Beam Technology Winterberg Straße 28 01277 Dresden Germany

**Keywords:** electrochemistry, energy storage, lithium-sulfur battery, solvent-free processing, sustainable binder

## Abstract

In the market for next‐generation energy storage, lithium‐sulfur (Li−S) technology is one of the most promising candidates due to its high theoretical specific energy and cost‐efficient ubiquitous active materials. In this study, this cell system was combined with a cost‐efficient sustainable solvent‐free electrode dry‐coating process (DRYtraec®). So far, this process has been only feasible with polytetrafluoroethylene (PTFE)‐based binders. To increase the sustainability of electrode processing and to decrease the undesired fluorine content of Li−S batteries, a renewable, biodegradable, and fluorine‐free polypeptide was employed as a binder for solvent‐free electrode manufacturing. The yielded sulfur/carbon dry‐film cathodes were electrochemically evaluated under lean electrolyte conditions at coin and pouch cell level, using the state‐of‐the‐art 1,2‐dimethoxyethane/1,3‐dioxolane electrolyte (DME/DOL) as well as the sparingly polysulfide‐solvating electrolytes hexylmethylether (HME)/DOL and tetramethylene sulfone/1,1,2,2‐tetrafluoroethyl‐2,2,3,3‐tetrafluoropropyl ether (TMS/TTE). These results demonstrated that the PTFE binder can be replaced by the biodegradable sericin as the cycle stability and performance of the cathodes was retained.

## Graphical abstract

**Green binder**: The polypeptide sericin is introduced as a binder for solvent‐free manufactured S/C cathodes via the DRYtraec® process. Their electrochemical performance is evaluated under lean electrolyte conditions at coin and pouch cell level, with ether‐ or sulfolane‐based electrolytes. It is demonstrated that the established polytetrafluoroethylene binder can be successfully replaced with the fluorine‐free sericin.

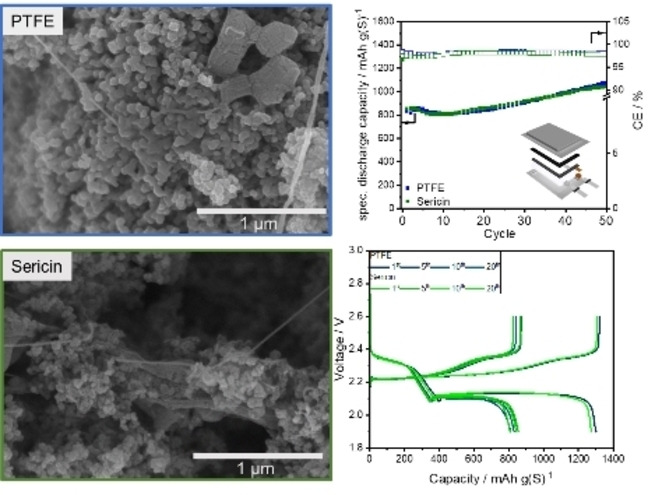

## Introduction

The increasing demand for mobile electrochemical energy storage is fueling the world‐wide demand for the precious and rare metals used in state‐of‐the‐art (SOTA) lithium‐ion batteries (LIBs) such as nickel (Ni) and cobalt (Co). Since their price continuously rises and they are often mined under unethical working conditions in politically unstable or autocratic countries, cost‐efficient and abundant alternative energy storage materials are required. Sulfur is a candidate that not only meets the requirement of ubiquitously available non‐toxic material, it also provides a theoretical specific capacity of 1672 mAh g(S)^−1^ and a theoretical energy density of 2500 Wh kg^−1^.^[1]^ So far, prototype cells with specific energies between 400–470 Wh kg^−1^ have been reported.^[2]^ Therefore, this cell system will be especially suited for aeronautical applications such as electric vertical take‐off and landing (eVTOL) and high‐altitude pseudo‐satellites (HAPS) where the weight of the energy storage system is a key factor.[^3^, ^4^]

Despite its obvious advantages, the Li−S system has not been commercialized yet due to some unresolved challenges, such as the polysulfide (PS) shuttle, corrosion of the lithium anode, and thereby a low cycle life. These challenges have been approached in the literature by several strategies, including the development of special electrolytes[^5^, ^6^, ^7^] and electrolyte additives,^[8]^ separators,^[9]^ protective anode coatings,^[10]^ separator coatings,^[11]^ and heteroatom‐doped polar carbon materials.[^12^, ^13^] The latter demonstrated their capability to improve the cycle stability by increasing the PS absorption and thereby restricting their mobility.^[14]^ Due to the increased interaction between the polar PS and the polar carbon surface, the mobility of the PS decreases. Furthermore, it was shown that doping the carbon with polar groups has a beneficial influence on the Li_2_S precipitation kinetics during the discharge.^[15]^ Additionally, it was demonstrated that the inclusion of polar additives increases the cathode wettability, as well as the Li_2_S precipitation.^[16]^

Another approach to increase the polarity of the cathode is the use of polymer binders with polar functional groups. It was demonstrated that the use of polar polymers as a binder or as cathode coating increases the capacity retention of those cells by retaining the formed PS in the cathode and decreasing the PS shuttle.[^17^, ^18^, ^19^] The PS retention is invoked by polar interactions as well as by forming H‐bridge bonds between the polar polymer and the PS.[^17^, ^19^, ^20^] Frequently, biopolymers such as polysaccharides^[21]^ or polypeptides^[22]^ are used as polar binders due to the abundance of hydroxylic and carboxylic groups in these polymers as well as their sustainability when compared to fluorinated binders.

**Table 1:** Binders applied for solvent‐free cathode manufacturing.

Binder	Molecular weight [g mol^−1^]
PTFE	10^7^–10^8[28]^
sericin	2800

These polar binders are mostly solvent‐processed in order to generate S/C cathodes: This coating route usually requires a cost‐ and energy‐intensive drying step. Depending of the solvent type, the drying and solvent recovery can make up to 9 % of the total production cost of a battery pack and more than 10 % of the total investment for a battery plant.^[23]^ This drying step is especially problematic for sulfur‐containing cathodes since the sulfur might sublime during the solvent evaporation.^[24]^ Additionally, it must be ensured that no traces of solvent remain in the electrode to avoid side‐reactions with other cell components. Thereby, solvent‐free electrode processing is an ideal approach to reduce the cost and carbon footprint of battery manufacturing.[^23^, ^25^] Numerous published solvent‐free electrode processing techniques depend on fluorine‐based polymers.[^26^, ^27^, ^28^] However, the production of these polymers is discussed in regard of health and environmental issues.^[29]^ Consequently, there is a need of a solvent‐free processable and fluorine‐free alternative. This alternative should be a biodegradable and sustainable as well as a polar polymer to increase the performance of the Li−S cell. Despite several possible alternatives such as carboxymethyl cellulose (CMC) or polyacrylic acid (PAA), which have been tested for their DRYtraec® processibility, no suitable substitute for the SOTA fluoropolymers was identified, as those polymers did not lead to coherent double‐sided coated electrode sheets. Another important criterion to be considered when selecting a possible alternative is its availability in industrially relevant quantities. A possible candidate is the polypeptide sericin, which is a by‐product of silk production obtained in large quantities, as its annual production volume is approximately 50 kt a^−1^.^[30]^ Moreover, this polypeptide was already used as a binder for slurry‐based LMNO cathodes, due to its strong adhesive properties invoked by its abundant polar groups.^[31]^

Herein, for the first time, the biodegradable polypeptide sericin is introduced as a binder in solvent‐free processed S/C cathodes and compared to polytetrafluoroethylene (PTFE) binder, which has been used for the DRYtraec® process so far.[^13^, ^27^, ^32^, ^33^] The surface morphology as well as the adhesion properties of these cathodes are characterized. Moreover, their wetting and swelling behavior is studied by confocal microscopy. The electrochemical performance of the sericin‐based cathodes is evaluated under lean electrolyte conditions on coin cell [Electrolyte/Sulfur: 5–7 μL mg(S)^−1^] and pouch cell level [E/S: 4.5 μL mg(S)^−1^]. The electrochemical evaluation of the cathodes is conducted in the SOTA 1,2‐dimethoxyethane/1,3‐dioxolane (DME/DOL) (DD) electrolyte and also in the sparingly PS solvating hexylmethylether (HME)/DOL (HD) and tetramethylene sulfone/1,1,2,2‐tetrafluoroethyl‐2,2,3,3‐tetrafluoropropyl ether (TMS/TTE) (TT) electrolytes. The resulting specific energies and energy densities of multi‐layered pouch cells depend on the respective electrolyte used and are stated in Table 2.

**Table 2:** Average specific energies and energy densities of the tested and discussed multi‐layered pouch cells.

Electrolyte	Average specific energy [Wh kg^−1^]			Average energy density [Wh L^−1^]		
	DD	HD	TT	DD	HD	TT
PTFE	211	221	180	435	450	405
sericin	220	172	183	458	391	415

## Results and Discussion

### Surface morphology

**Figure 1:**
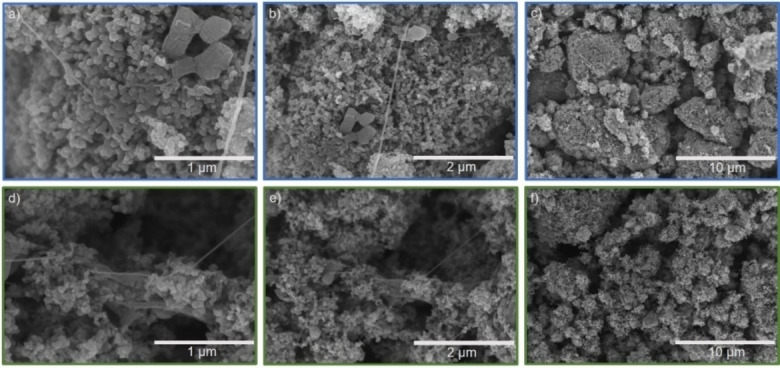
SEM images of (a–c) PTFE‐based and (d–f) sericin‐based dry‐film cathodes.

In previous publications, S/C cathodes based on different carbon materials were all prepared, following the same solvent‐free approach, with the established PTFE binder.[^13^, ^27^, ^32^, ^33^] Such S/C cathodes based on the SOTA PTFE binder are depicted in Figure 1a–c. In Figure 1b,c PTFE fibrils are visible. These fibrils are characteristic of the PTFE binder and are crucial for the cathode film formation, as well as for the cohesion of the cathode itself. The fibrils connect the single S/Ketjenblack (KB) agglomerate particles by forming a spiderweb‐like network, as visible in Figure 1b,c. These fibrils are formed by shearing the PTFE spheres (Figure S1a). The length of these fibrils can exceed 100 μm while their thickness can attain several micrometers, as depicted in Figure S2a.

Similar to PTFE, sericin also forms fibrils during the shearing of the material. In contrast to PTFE, sericin is not present as spheres but as platelets, as shown in Figure S1b. The fibrils formed by sericin are both shorter and thinner than the ones formed by PTFE. The smaller size of the sericin fibrils could be induced by the lower molecular weight of this polymer. As shown in Figure 1d, these fibrils are micrometer‐scaled and connect the single nanoparticles. No micrometer‐scaled sericin fibrils can be observed in Figure S2b. Comparable to PTFE, sericin also connects the single carbon agglomerates by forming a spiderweb‐like structure, as shown in Figure 1e.

In the following, the resulting cathodes are investigated by dynamic swelling analysis via confocal microscopy which is not only a tool to investigate the swelling and wetting behavior of the cathodes but can also be applied to evaluate the capability of the binder to ensure the cohesion of the cathode as well. Since it was demonstrated that the dry‐film cathodes swell and expand following the contact with ether‐based electrolytes,^[27]^ it is important to evaluate if the binder can withstand the mechanical stress applied to the cathode during the swelling.

**Figure 2:**
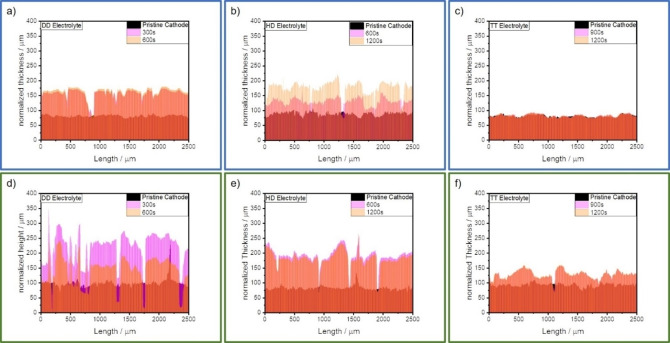
Height profiles of (a–c) PTFE‐ and (d–f) sericin‐based cathodes after10 μL of (a,d) DD, (b,e) HD, and (c,f) TT electrolyte is applied. The pristine electrode thickness is normalized on the actual thickness of the dry‐film electrode coating.

First, the pristine cathode surface is studied via confocal microscopy. Subsequently, 10 μL of the respective electrolyte is applied on the cathode surface to induce and/or to evaluate the swelling of the cathodes. The swelling of the cathodes is subsequently monitored after certain time intervals. The dimension of the monitored area is 2500×2000 μm^2^. In Figure 2a, the height profiles of the PTFE‐based cathode which was wetted with DD electrolyte are depicted. The initial average thickness of this cathode is 82 μm, while the density is 0.5 g cm^−3^. The thickness is increased to 162 μm after 600 s, corresponding to an increase by 98 %. Thereby, the cathode density is reduced to 0.26 g cm^−3^. As well as in the height profiles, big gaps can be observed in the surface of the cathode where the electrolyte was deposited in Figure S3a. These gaps indicate that PTFE is not capable to withstand the stress during the swelling of the cathode. The length of these gaps can exceed one millimeter, while their width is only micrometer‐scaled. No formation of fissures can be observed on the pristine surface of the cathode, which was not wetted by the electrolyte.

Figure 2d shows the height profiles of a DD‐wetted sericin‐based cathode. The initial average thickness and density of this cathode is 96 μm and 0.44 g cm^−3^, respectively. After 600 s, the thickness of the cathode is increased to 188 μm whereby the density is reduced to 0.23 g cm^−3^. This translates to a thickness increase of 98 % and a density decrease of 48 %, respectively. These results are comparable to the ones obtained with the PTFE‐based standard cathode. The cathode exhibits an increased swelling after 300 s followed by a reduction of the thickness later. This could be related to a faster wetting of the cathode due to the higher polarity of the sericin binder. As with the PTFE‐based cathode, the swelling inflicted fissures to the sericin‐based cathode. The dimensions of these gaps are comparable to the ones formed in the reference PTFE‐cathode.

The swelling dynamics of both cathodes are also evaluated with two other sparingly polysulfide‐solvating electrolytes (SPSEs), HD and TMS/TTE. For HD electrolyte, as depicted in Figure 2b,d, the time interval until the first measurement had to be increased due to the slower cathode wetting of the HD electrolyte. 1200 s after the electrolyte is deposited on the standard cathode its thickness has increased by 115 %, from 89 to 191 μm. The cathode density decreases by 53 %, from 0.47 to 0.22 g cm^−3^, respectively. The thickness of the sericin‐based cathode increases from 83 to 181 μm, causing an expansion by 118 %. The corresponding cathode density is reduced by 54 %, from 0.5 to 0.23 g cm^−3^, respectively. As described above regarding the DD electrolyte, a faster wetting of the sericin‐based cathodes is observed. The surfaces of both cathodes exhibit a formation of gaps after swelling in HD electrolyte, as depicted in Figure S3b,e. As for the DD electrolyte, the formed gaps are micrometer to millimeter scaled and appear only in the area wetted by the electrolyte.

As depicted in Figure 2c,f, both cathodes are wetted with the sulfolane‐based TT electrolyte, to investigate their swelling behavior in this sparingly PS solvating electrolyte. The PTFE‐based cathode expands only by 2 % as demonstrated in Figure 2c. The thickness increases from 81 to 83 μm, respectively. The cathode density is marginally decreased from 0.51 to 0.50 g cm^−3^. In contrast, to the standard cathode, the sericin‐based cathode exhibits a height increase of 37 % while swelling. The active layer increases from an average thickness of 93 to 127 μm, and consequently, the density is decreased from 0.45 to 0.33 g cm^−3^. The sericin‐based cathode in Figure S3f shows micrometer‐scaled fractures after the swelling and the associated expansion, whereas such behavior was not observed for the reference cathode in Figure S3c. The absence of fissures in the PTFE‐based cathode is related to its slight expansion and therefore to the low resulting mechanical stress. Moreover, it can be stated that the swelling and expansion of the electrodes is less pronounced with the sulfolane‐based TT electrolyte when compared with the ether‐based electrolytes DD and HD.

It can be concluded that both PTFE‐ and sericin‐based cathodes expand in ether‐based electrolytes and have a comparable increase in thickness. Furthermore, both binders are not able to guarantee the integrity and/or density of the cathode coating while swelling/expanding. Since the binder content is only 3 wt %, this is by no means unexpected. Therefore, it can be concluded that the swelling of both cathodes is comparable in ether‐based electrolytes and that the influence of the binder systems is limited. Contrary to the results obtained with the ether‐based electrolytes, a difference in swelling can be observed indeed for the TT electrolyte. The PTFE‐based cathodes experienced only a minor expansion, whereas the sericin‐based one expanded by 37 % after the TT uptake. As the PTFE‐based cathode did not expand no mechanical stress is formed in the cathode which could lead to damage to the structure of the cathode. The different swelling behavior of the ether‐based electrolytes and the sulfolane‐based one likely results from the dissimilar chemical structure of the electrolyte solvents as well as to the different electrolyte properties such the surface tension, wetting, and density of those electrolytes. Further, it was demonstrated that the viscosity of electrolytes with high salt concentrations has a major impact on the wetting of LiNi_0.5_Mn_0.3_Co_0.2_O_2_ (NMC532)‐based electrodes.^[34]^ Therefore, the swelling behavior might also be affected by the viscosity of the electrolyte. As the chemical structure of both ether‐based electrolytes and their dynamic viscosities are comparable (DD: 2 mPa s; HD: 5.5 mPa s),[^33^, ^35^] their swelling behavior is quite similar as well. The less pronounced swelling of the sulfolane‐based TT electrolyte is ascribed to its higher dynamic viscosity of 24 mPa s.^[35]^ The stronger swelling of the sericin‐based electrode in the TT electrolyte compared to the PTFE‐based reference could by induced by a more pronounced interaction between the cathode and electrolyte. This might be invoked by an increased cathode polarity, due to the polar surface groups of the binder. Hence, an extensive study on the complex interaction between the respective electrolytes and the prepared and presented dry‐film electrodes is beyond the scope of this work.

### Mechanical characterization of the cathodes

The adhesion of both cathodes to the current collector is evaluated by 180° peel‐off tests. Therefore, adhesive tape is attached to the cathode surface, and the area of the adhesive joint is 1 cm^2^. The instrument measures the applied force as the cathode/tape joint is extended at a constant speed. The maximum force is reached as soon as the adhesive joint fails. The maximum adhesion force of the electrode coating is therefore the force applied at the point of failure.

**Figure 3:**
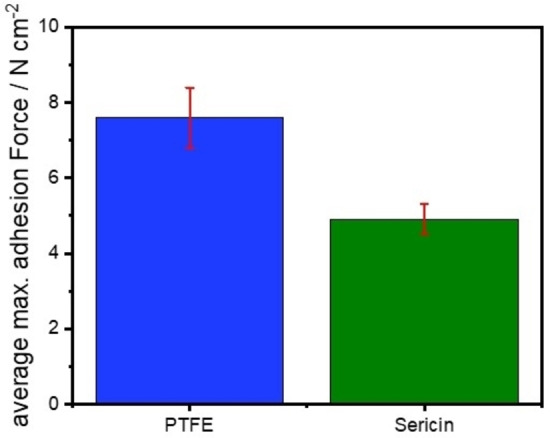
Average adhesion forces of PTFE‐ and sericin‐based dry‐film coatings to the aluminum current collector.

As depicted in Figure 3, the adhesion force of the PTFE‐based cathode is higher than of the sericin‐based one. The maximum adhesion forces are 7.6 and 4.9 N cm^−2^, respectively. These adhesion forces are average values obtained by a three‐fold determination. The average adhesion force of the sericin‐based cathodes is 35 % lower than the one of the PTFE‐based standard dry‐film cathodes. The better adhesion of the PTFE‐based cathodes could be explained by its long‐distance fibrils, which connects the cathode layer better with the current collector. Furthermore, the lower molecular weight of the sericin might invoke the lower adhesion of these cathodes as it was demonstrated in the literature that the increase in molecular weight of the polymer binder has a beneficial impact of the electrode adhesion.^[36]^ Nonetheless, the adhesion of the sericin‐based cathode is sufficient to ensure roll‐to‐roll (R2R) processing and handling. In addition, it must be noted that the sericin material has not been optimized yet for its application as binder in electrodes. The PTFE material, in contrast, is well adapted for this purpose.

### Electrochemical analysis in coin cells

The electrochemical performance of both cathodes is evaluated on coin cell level vs. Li/Li^+^ with both ether‐based electrolytes as well as with TT electrolyte. An E/S ratio of 7 μL mg(S)^−1^ is applied for the coin cell testing with DD electrolyte, while the evaluation with both SPSEs is carried out under a lean electrolyte regime [E/S=5 μL mg(S)^−1^].

**Figure 4:**
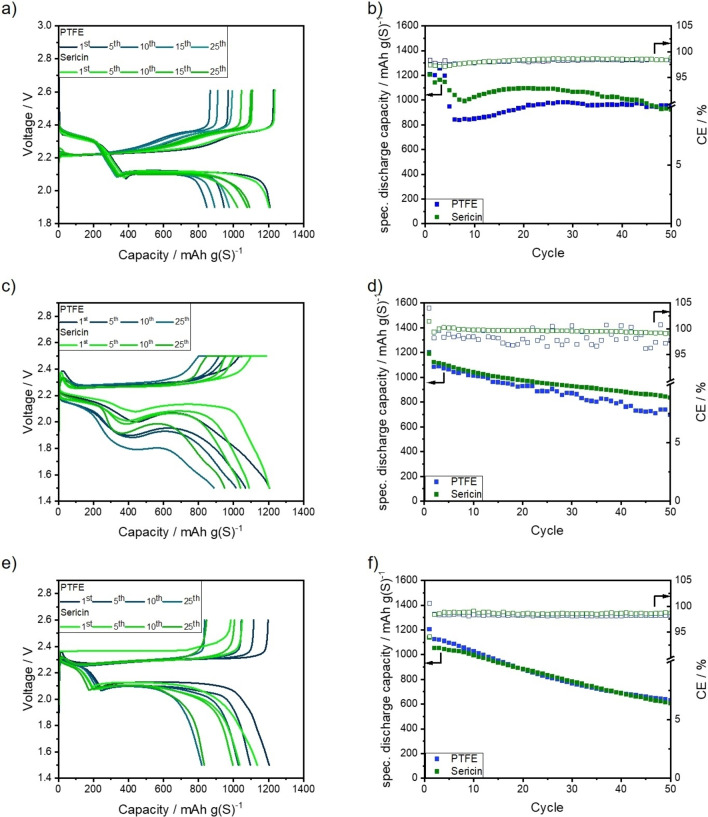
Voltage profiles and galvanostatic cycling stability on coin cell level of PTFE‐ and sericin‐based cathodes in (a,b) DD [E/S=7 μL mg(S)^−1^], (c,d) HD [E/S=5 μL mg(S)^−1^], and (e,f) TT [E/S=5 μL mg(S)^−1^] electrolyte.

Both cathodes achieve an initial discharge capacity of approximately 1205 mAh g(S)^−1^ in DD electrolyte at coin cell level. The amount of sulfur that is addressed during the first discharge plateau at 2.3 V is also comparable. The first plateau in DD electrolyte is linked to the conversion of S_8_ to long‐chain PS such as Li_2_S_8_ and Li_2_S_6_.^[37]^ Both cathodes yield a capacity of approximately 390 mAh g(S)^−1^, as depicted in Figure 4a. The end of this plateau is characterized by the “voltage dip”, where a maximum overpotential is reached and the nucleation of solid Li_2_S begins. The increase in overpotential is invoked by an increase of PS concentration in the electrolyte and thereby by a decrease in ionic conductivity and an increase of electrolyte viscosity. The fast rise in PS concentration can be appointed to the disproportionation of dissolved PS to short‐chain PS, such as Li_2_S_4_.^[38]^ During the second discharge plateau at 2.1 V, the dissolved PS are further reduced to short‐chain PS, for example Li_2_S_3_. The short‐chain PS are then further reduced to insoluble Li_2_S_2_ and Li_2_S. Thereby, the concentration of dissolved PS in the electrolyte is reduced again.^[39]^ The precipitated Li_2_S forms a non‐uniform and highly porous layer on the surface of the electrode. It was demonstrated by small‐angle neutron scattering (SANS) that the diameter of the formed Li_2_S particles is below 2 nm.^[40]^ Simulations by Danner and Latz and Ren et al. also indicate a formation of nano‐scaled Li_2_S particles during the discharge.[^15^, ^41^] Moreover, they revealed that the particle size of the formed solid depends on the applied C‐rate and that a formation of smaller particles is favored at higher C‐rates. These findings are validated by operando X‐ray diffraction (XRD) experiments.^[42]^ Whereas another study suggests that the formed Li_2_S layer consists of nanometer to micrometer‐scaled particles.^[43]^ Due to the depletion of dissolved PS at or in the cathode and the increasing length of the diffusion pathways (by the formation of a Li_2_S‐layer) the cells’ overpotential is rapidly increasing at the end of the second discharge plateau and thereby terminating the discharge reaction.^[43]^ Furthermore, it has to be taken into account that the conductive carbon surface is passivated by the insulating Li_2_S layer formed during the discharge.^[41]^

Both cathodes deliver discharge capacities of more than 800 mAh g(S)^−1^ in the second discharge plateau. During the charge, this reaction cascade is reversed, and macroscopic crystalline sulfur is formed at the end of the charging step.[^40^, ^44^] During the next 10 cycles, a fast degradation of discharge capacity can be observed for both cathodes, whereas the capacity fading is less pronounced for the sericin‐based cathodes. This reduction in discharge capacity can solely be appointed to a reduction of the second discharge plateau. In the fifth cycle, the sericin‐based electrode still yields a discharge capacity of 1077 mAh g(S)^−1^, whereas the PTFE‐based one delivers a discharge capacity of 946 mAh g(S)^−1^. After 10 cycles, the discrepancy in discharge capacity between the sericin‐ and PTFE‐based increases further. The cathodes yield after the 10th cycle capacities of 1022 and 847 mAh g(S)^−1^, respectively. An increase in discharge capacity can be noticed afterwards. This behavior is already known from KB‐based dry‐film cathodes as the carbon black particle network has a strong swelling tendency.[^13^, ^27^] After 25 cycles, the capacity of the PTFE‐based capacity increased to 975 mAh g(S)^−1^, whereas the sericin‐based cathode retains a capacity of 1091 mAh g(S)^−1^. The less pronounced reduction of the second discharge plateau of the sericin‐based cathode could be attributed to an improved Li_2_S deposition due to the polar surface groups of the binder, as it was demonstrated that an increase in surface energy/polarity leads to the formation of less and bigger Li_2_S agglomerates instead of a porous film.^[15]^ In addition, electrolyte wetting might be enhanced, as well. After 50 cycles both cathodes yield capacities of more than 900 mAh g(S)^−1^, as shown in Figure 4b. After 50 cycles, the degradation of the cell capacity can be mainly appointed to the degradation of the lithium anode.^[45]^ The sericin‐based cathode demonstrated a more stable performance than the PTFE‐based cathodes during the first 50 cycles in DD electrolyte at coin cell level.

Moreover, both cathodes were tested with the SPSE HD as shown in Figure 4c,d. As depicted in Figure 4c, both cathodes achieve comparable discharge capacities in the first cycle. The initial discharge capacities of the PTFE‐ and sericin‐based cathodes are 1205 and 1203 mAh g(S)^−1^, respectively. Two discharge plateaus can be distinguished for both cathodes. The first discharge plateau in HD electrolyte is linked to the formation of short‐chain PS. According to the literature, the utilized sulfur is mainly converted to Li_2_S_4_ during the first discharge plateau.^[7]^ Within this activation step, first capacities of 446 and 441 mAh g(S)^−1^ are obtained for the PTFE‐ and sericin‐based cathodes.

Although the capacities obtained are similar, the PTFE‐based cathode has a higher overpotential during discharge. At the end of the first discharge plateau, the PTFE‐based cathode features an overpotential of 0.1 V. This overpotential is constant during the second plateau. The overpotential of the second plateau might be a hint that the Li_2_S formation and deposition is hampered. The lower overpotential in the 2nd plateau of the sericin‐based cathode could be appointed to the improved deposition of the Li_2_S during discharge by polar groups or better wettability of the binder.^[15]^

After 10 cycles, the sericin‐based cathode retains a discharge capacity of 1038 mAh g(S)^−1^, while PTFE‐based cathode shows a capacity of 1013 mAh g(S)^−1^. Similar to the first cycle, the PTFE‐based cathode exhibits an overpotential of 0.1 V at the end of the first plateau. After 25 cycles, the PTFE‐based cathode achieves 886 mAh g(S)^‐1^, whereas the sericin cathode delivers a capacity of 947 mAh g(S)^‐1^). The strong capacity degradation of the PTFE‐based cell is invoked by a rapidly increasing overpotential during the second plateau.

After 50 cycles at C/10, the sericin‐based cathode still delivers a discharge capacity of 836 mAh g(S)^−1^, while the PTFE‐based cathode achieves a capacity of only 695 mAh g(S)^−1^, as in Figure 4d. Moreover, it has to be noted that the average coulombic efficiency (CE) of the sericin‐based cathode during the first 50 cycles is higher than the one of the PTFE‐based cathodes. The average CEs are 99.2 and 98.3 %, respectively. The slower capacity degradation of the sericin‐based cathode might be caused by an improved Li_2_S deposition behavior. Nonetheless, the influence of the binder on the surface polarity and/or wetting behavior of the electrolyte should not be overestimated since its fraction of the cathode is only 3 wt %.

As stated previously, both cathodes are also tested with TT electrolyte at coin cell level. In Figure 4e, the voltage profiles of both cells are shown. The initial capacity of the PTFE‐based in the respective electrolyte is 1201 mAh g(S)^−1^, whereas the capacity of the sericin‐based cathode is 1136 mAh g(S)^−1^. This slight deviation could be explained by a reduced sulfur utilization in the first plateau at 2.3 V. The first plateau of the discharge slope in TT electrolyte is short due to its restricted polysulfide solubility and reduced formation of long‐chain polysulfides. Therefore, less than 20 % of the initial discharge capacity of both cathodes is generated in the first discharge plateau.^[6]^

After 25 cycles, both cathodes deliver comparable discharge capacities of approximately 830 mAh g(S)^−1^. After 50 cycles the PTFE‐based cathodes deliver 633 mAh g(S)^−1^, whereas the sericin‐based one achieves 616 mAh g(S)^−1^, as depicted in Figure 4f. Both cathodes show the linear capacity degradation characteristic for TT electrolyte during cycling.[^6^, ^46^] The first 50 cycles demonstrate that the average CE of the sericin‐based cathode is comparable to the PTFE‐based cathode: The average CEs are 98.6 and 98.3 %, respectively. Therefore, it can be concluded that both cathodes demonstrate similar behavior in the respective electrolyte.

It can be summarized that a slight influence of the binder on the performance of the cathodes at coin cell‐level can be observed. However, this impact should not be overestimated, especially when SPSEs are employed. This could be assigned to the fact that the weight fraction of the binder in the cathode is comparably low. In DME/DOL electrolyte, however, the difference between sericin‐ and PTFE‐based binder is more pronounced, and a higher discharge capacity in the second plateau could be reached by the cathodes with the polar sericin binder. If the PS solubility is not restricted, the polar groups of the binder have a higher impact on the PS migration out of the cathode. In addition, the swelling behavior is also increased. Furthermore, the polar surface groups could improve the Li_2_S precipitation and promote the formation of a less dense/tortuous passivation layer. Such effects should be evaluated on pouch cell level as pressure distribution in coin cells during cycling can be changed and are not controllable.

### Electrochemical evaluation in pouch cells

In order to validate the results obtained at coin cell level, five‐layered pouch cells were assembled and electrochemically evaluated under lean electrolyte conditions [E/S=4.5 μL mg(S)^ 1^], and the cells were cycled under constant uniaxial pressure of 0.31 MPa. The gravimetric as well as volumetric energies of the respective cells are listed in Table 2.

**Figure 5:**
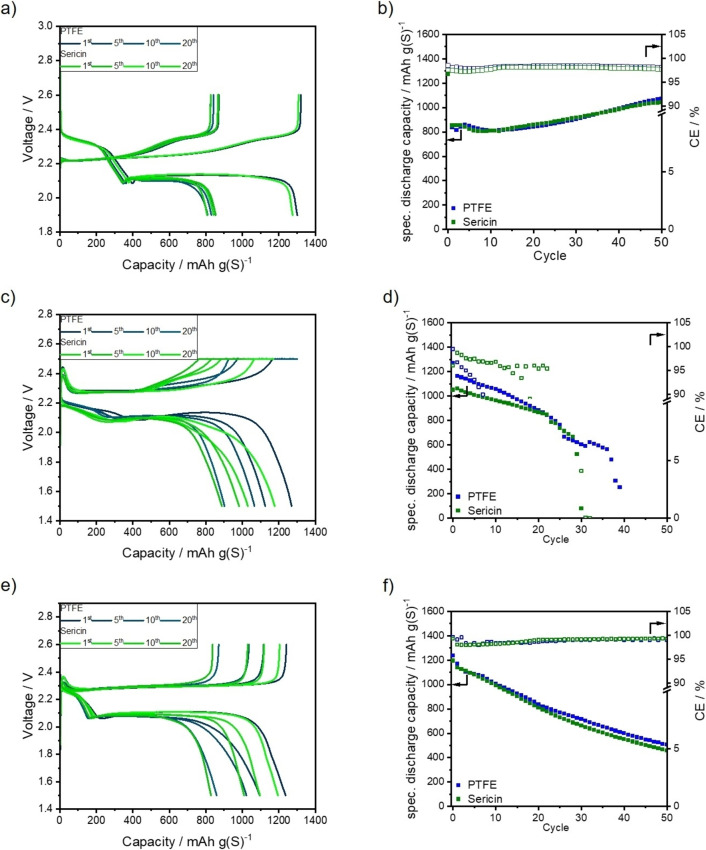
Voltage profiles and galvanostatic cycling stability on pouch cell level of PTFE‐ and sericin‐based cathodes in (a,b) DD, (c,d) HD, and (e,f) TT electrolyte. An E/S ration of 4.5 μL mg(S)^−1^ was applied. The same testing procedure as for the coin cells was conducted.

The performance of the pouch cells filled with DD electrolyte are shown in Figure 5a,d. Initially, the standard cathode delivers an initial discharge capacity of 1298 mAh g(S)^−1^, whereas the sericin‐based cathode yields comparable 1276 mAh g(S)^−1^. The voltage profiles of the initial discharge exhibit both characteristic plateaus at 2.3 and 2.1 V, as illustrated in Figure 5a. The sulfur utilization during the first plateau is comparable for both cathodes. As at coin cell level, both pouch cells show a fast capacity degradation during the first cycles. In the fifth discharge, the PTFE cell achieves a capacity of 854 mAh g(S)^−1^, while the capacity of the sericin‐based cathode is 843 mAh g(S)^−1^. Again, this fast decline can be attributed to a shortening of the second discharge plateau. Contrarily to the coin cell results, no obvious improvement can be observed using sericin as a binder. After 10 cycles, the capacity increases again, as the second discharge plateau is more utilized. Discharge capacities of 853 and 830 mAh g(S)^−1^ were yielded from the sericin and PTFE cells after 20 cycles. After 50 cycles, both cells still achieve discharge capacities of more than 1050 mAh g(S)^−1^, as depicted in Figure 5b. During the first 50 cycles, the average CE of the PTFE‐based cells is 98.2 %, whereas the CE of the sericin‐based cell is 97.9 %. It was reported in our previous study that the performance of solvent‐free processed S/C cathodes in DME/DOL‐filled pouch cells strongly depends on the applied external pressure and thereby on the compaction or density of the cathodes. Therefore, further pouch cells without external pressure application were cycled with DD electrolyte.^[33]^ The discharge capacity of the sericin‐based cathode is increased by the elimination of the external pressure, as depicted in Figure S4a where an initial discharge capacity of 1328 mAh g(S)^−1^ is obtained. This increase can be ascribed to a prolongation of the 2^nd^ discharge plateau and thereby on an improved precipitation of the solid discharge products, as depicted in Figure S4a. The sulfur utilization was not affected by the reduction of the external pressure, since the first discharge plateau is not altered. Furthermore, the capacity degradation in the first cycles is less pronounced for the pressure‐less tested cells as for the ones tested under external pressure. In the first cycles, both non‐pressurized cells achieved discharge capacities of approximately 1200 mAh g(S)^−1^, while their pressurized counterparts yielded capacities of less than 900 mAh g(S)^−1^, as illustrated in Figure S4c. In the subsequent cycles, no significant impact of the used binder on the performance of the pressure‐less tested cells is observed. This is in agreement with the results acquired by the cells tested under pressure. Both cells fail after the 36 cycles. In the 35th cycle, the sercin‐based cell retained a discharge capacity of 928 mAh g(S)^‐1^, while the PTFE‐based one retained 934 mAh g(S)^−1^. The accelerated capacity degradation and earlier failing of the non‐pressurized cell could be assigned to an increased degradation of the lithium anode. The stronger degradation of the lithium is caused by an extensive stress on the anode as more lithium had to be stripped and plated due to the higher capacities and transferred charge. Additionally, it must be considered that also more electrolyte is consumed, since more fresh lithium surface, that has to be covered by solid electrolyte interphase (SEI), is formed in every cycle, if more lithium is moved.^[33]^

To facilitate the pouch cell characterization and reduce the required time for cycling. The charging of the HD filled pouch cells was conducted with 0.1 C instead of 0.05 C. Thereby, a stark contrast between coin cell and pouch cell results can be observed for the HD‐filled cells. At coin cell level, the sericin‐based cathodes exhibited a lower overpotential during the second discharge plateau and a comparable discharge capacity, whereas on pouch cell level no significant overpotential is observed in the second plateau, as shown in Figure 5c, although a higher overpotential is observed for the sericin‐based cell during the first discharge plateau. While a capacity of approximately 430 mAh g(S)^−1^ is measured for the PTFE cathode, only 343 mAh g(S)^−1^ is retained from the sericin cathode during the first plateau. In the subsequent cycles, this difference is reduced, whereas the overpotential of 0.05 V is retained. After 10 cycles, the capacity of the sericin‐based cell is 963 mAh g(S)^−1^, whereas a discharge capacity of 982 mAh g(S)^−1^ was yielded by the PTFE standard cathode. In the subsequent cycles, the sericin‐based cells show lower capacity degradation than the standard cells. Therefore, comparable discharge capacities of approximately 860 mAh g(S)^−1^ were achieved by both cathode systems in the 20th cycle. Hence, after 23 cycles, a sudden drop of discharge capacity and afterwards an increased capacity degradation can be observed for the sericin‐based cell. The PTFE reference cell also shows a drop in capacity, after 25 cycles, and ultimately fails thirteen cycles after. The fast degradation of the tested HD‐based pouch cells can be appointed to the increased current during the charge, as it was demonstrated in a previous publication that more than 600 mAh g(S)^−1^ could be reached after 40 cycles if a charge current of 0.05 C was applied.^[33]^

As demonstrated in coin cells, the influence of the binder on the electrochemical performance of TT‐filled cells is limited also in pouch cells as depicted in Figure 5e. The initial discharge capacity of the PTFE based electrode is 1236 mAh g(S)^−1^ and thereby slightly higher than the one of the sericin‐based cathode [1198 mAh g(S)^−1^]. As at coin cell level, the initial capacity of the PTFE‐based cathode is slightly higher than the one of the sericin‐based one. Furthermore, it can be noted that the TT‐based pouch cells yield higher initial capacities than the corresponding coin cells. The voltage profiles shown in Figure 5e are comparable to the ones obtained in coin cells, depicted in Figure 4e, as two discharge plateaus at 2.2 and 2.1 V are distinguishable. In the fifth cycle, both cathodes yield a discharge capacity of 1097 mAh g(S)^‐1^. The long‐term stability of both cathodes is also comparable, as illustrated in Figure 5f. Both cells exhibit a nearly linear capacity degradation. This degradation behavior could be also observed in coin cells. After 50 cycles the sericin‐based cathodes still yield a discharge capacity of 469 mAh g(S)^−1^, whereas the PTFE reference cathode retains 498 mAh g(S)^−1^. The average CE of both cathodes during the first 50 cycles is 98.8 %.

The evaluation of the cathodes on pouch cell level demonstrated that the influence of the applied binders on the performance of the cathodes is limited, independent from the electrolyte used. This is in contrast to the results attained in coin cells. These dissimilarities could arise from a divergent compaction of the cathodes in the two cell types. A particularly pronounced difference between the cell types was observed for DD‐filled cells. As previously demonstrated this electrolyte is especially affected by a change of cathode density due to its sulfur conversion mechanism, this difference in performance between the pouch and the coin cells might by invoked by varying cathode compression.^[33]^ Whereas the external pressure on the cathodes can be precisely controlled in pouch cells, this is not possible in coin cells, where the exact pressure exerted by the cell housing on the cathode is not discernable. Further, it was demonstrated in another publication that the density of the dry‐film cathodes can be affected by swelling after electrolyte uptake. Thereby, the density of those cathodes is altered.^[27]^ While the swelling of the cathodes can be well‐controlled in pouch cells this is not possible in coin cells as the cathodes could swell into the large dead volume of the coin cells. The higher dead volume of the coin cells is another parameter, which makes it difficult to compare results obtained in different cell types, since these cells have to be filled with more electrolyte. This, in combination with a higher excess of lithium contributes to the increased lifetime of the tested coin cells. Thus, it can be concluded that all findings obtained by coin cell testing should be validated in pouch cells, to avoid over interpretation of results achieved under unrealistic and uncertain conditions.[^4^, ^47^] Furthermore, it was demonstrated that the special behavior of the dry‐film cathodes in DD‐filled pouch cells, which was already observed, is linked to the applied pressure. However, the underlying mechanism is beyond the scope of this work and will be discussed further in another publication.

Summarizing, the pouch cell results demonstrate that both employed binders, sericin and PTFE, have no significant influence on the performance of the cathodes on pouch cell level, regardless of the used electrolyte. Therefore, it can be concluded that PTFE could be successfully replaced by a biodegradable and sustainable biopolymer as a binder in solvent‐free processed Li−S battery cathodes.

## Conclusion

Herein, the biodegradable polypeptide sericin was introduced for the first time as a binder in a solvent‐free sulfur/carbon cathode coating process. The R2R processed S/C cathodes were morphologically and mechanically characterized. It was demonstrated that the used binder has only a minor influence on the swelling and wetting properties of the respective cathode if ether‐based electrolytes were applied. Additionally, the polytetrafluoroethylene (PTFE)‐based standard and the sericin‐based cathodes were electrochemically evaluated in coin as well as in pouch cells. The electrochemical characterization was conducted with the state‐of‐the‐art 1,2‐dimethoxyethane/1,3‐dioxolane (DME/DOL) system and with two types of sparingly polysulfide solvating electrolytes: hexylmethylether (HME)/DOL and the sulfolane‐based tetramethylene sulfone/1,1,2,2‐tetrafluoroethyl‐2,2,3,3‐tetrafluoropropyl ether (TMS/TTE) system. While the coin cell results suggested that the polar groups of the binder improve the performance of the cathode in the highly polysulfide‐solvating DME/DOL electrolyte, such improvement could not be identified in the pouch cell study. The results obtained in pouch as well as in coin cells show that neither significant improvement nor deterioration of cell performance by the application of sericin could be observed. Consequently, sericin is prospectively a promising biodegradable fluorine‐free candidate in order to replace PTFE.

Another advantage of sericin is its electrochemical stability at lower potentials/voltages, as it was used as a protective lithium anode coating.^[48]^ This qualifies sericin as a binder also for solvent‐free processed anodes, whereas PTFE is not suited as an anode binder due to decomposition at lower voltages.^[49]^

## Experimental Section

### Preparation of sulfur/carbon cathodes

To prepare the sulfur/carbon composite, pristine sulfur (Sigma Aldrich, >99.5 %) and Ketjenblack (KB) EC600JD (AkzoNobel) were blended in a centrifugal mill (ZM200 Retsch GmbH) in a ratio of 1.6 to 1. Subsequently, the infiltration was carried out at 155 °C under air. Next, 3 wt % of the respective binder was mixed with the composite in a blade mill. The applied binders are listed in Table 1.

The as‐prepared electrode composites were used to manufacture S/C cathodes solvent‐free via the DRYtraec® process. The preparation method is described elsewhere.^[50]^ This process allows the production of electrodes by shear forces induced by different rotational speeds of counter‐rotating calender rollers. These electrode films were immediately transferred to primer‐coated aluminum current collector current (15 μm aluminum). The composition of the prepared single‐sided cathode was S/KB/binder=60 : 37 : 3. The as‐prepared electrodes had a sulfur loading of 2.5±0.2 mg cm^−2^ and an electrode density of 0.5±0.1 g cm^−3^. These average values were derived from a minimum of ten individual cathode discs. The cathode thickness was determined with an FD‐50 thickness gauge (Schmidt control instruments).

### Mechanical characterization

An adhesive joint (area of this bond was 1 cm^2^) was formed between a double‐sided adhesive tape (Scotch Crystal Clear 600) and the respective S/C cathodes. Subsequently, the adhesive joints were compressed with uniaxial hydraulic pressure (600 kPa; RT; LaboPress P200S VOGT Labormaschinen GmbH). The adhesion force of these samples was determined with a high‐precision Digital Force Gauge (FH2, Sauter GmbH, Germany) in a 180° peel setup. The adhesion test setup was inspired by DIN EN 28510‐1. A constant displacement rate of 60 μm s^‐1^ was applied for testing and the force was monitored. The maximum adhesion forces were averaged by three‐fold determination at least.

### Electrochemical analysis in coin cells

Prior to coin cell (CR2016, MTI Corp.) assembly the electrode discs (diameter: 15 mm) were vacuum‐dried at 50 °C for 1 h. The cell assembly was conducted in an argon‐filled glovebox (MBraun, conditions: <0.1 ppm O_2_ and H_2_O). A polyethylene (PE) separator (thickness: 12 μm; diameter: 19 mm) and a Li Chip (thickness: 250 μm; diameter: 16.5 mm; MTI Corp.) were used together with a stainless‐steel spacer (thickness: 1000 μm; diameter: 16.3 mm; MTI Corp.) to assemble the coin cell. The electrolyte was added prior to sealing (sealing pressure: 3.5 MPa). Three different electrolytes were applied for cell testing. First, a mixture of DME (battery‐grade, Gotion, Inc) and DOL (battery grade, Gotion, Inc) (*v*/*v* 1 : 1) with 1 m Lithium bis(trifluoromethanesulfonyl)imide (LiTFSI, Gotion,Inc.) and 0.5 m LiNO_3_ (Alfa Aesar). An E/S ratio of 7 μL mg(S)^−1^ was applied for this electrolyte. Second, a mixture of HME (TCI Chemicals Deutschland GmbH) and DOL (*v*/*v* 9 : 1) with 2 m LiTFSI. Third, a mixture of TMS (Kishida Chemical Co., Ltd.) and TTE (Daikin Chemical Europe GmbH) (*v*/*v* 1 : 1) with 1.5 m LiTFSI. An E/S ratio of 5 μL mg(S)^−1^ was applied for both electrolytes. The preparation of both electrolytes was reported elsewhere.[^6^, ^7^] The DD‐filled cells were tested with a constant C‐rate of 0.1 C in a voltage window between 1.9 and 2.6 V vs. Li/Li^+^, where 1 C=1672 mAh g(S)^−1^. The first cycle of the HD‐based was measured in a voltage range from 1.5 to 2.5 V vs. Li/Li^+^ with a C‐rate of 0.05 C. Subsequent cycles were discharged with 0.1 C and charged with 0.05 C, whereas at the end of the charging step a constant voltage step at 2.5 V was applied until a current of 1/50 of the nominal capacity was gained. Similar to the HD cells, a C‐rate of 0.05 C was applied for cycling the first cycle of the TT‐filled cells; afterwards, these cells were cycled with 0.1 C in a voltage domain from 1.5 to 2.6 V. The electrochemical characterization was conducted at a constant laboratory temperature of 25±1 °C. The galvanostatic cycling measurements were performed with a BASYTEC CTS system (BaSyTec GmbH).

### Electrochemical evaluation on pouch cell level

Double‐sided cathodes were prepared following the same procedure described earlier. The R2R processed cathode coils were cut by remote laser cutting to dimensions of 71×46 mm^2^. The average sulfur loading and density of the double‐sided were 2.6 mg cm^−2^ and 0.8 g cm^−3^, respectively. Subsequently, five cathodes were stacked with PE separator and double‐sided lithium metal foil (71×46 mm^2^; 2×50 μm; CEL China Energy Lithium Co., Ltd) to assemble five‐layered pouch cells. The cells were then filled with the respective electrolyte types described above. An E/S ratio of 4.5 μL mg(S)^−1^ was applied. The electrochemical characterization of the five‐layered pouch cell was carried out at a constant temperature of 25±1 °C with a BASYTEC CTS system (BaSyTec GmbH). While testing a uniaxial pressure of 0.31 MPa was applied on the cells, by a pneumatic pressure control system (Fraunhofer IWS). As for the respective coin cell tests, the characterization was conducted as a two‐fold determination. The same cycling parameters were applied for the DD and TT‐filled pouch cell tests. The initial cycle of the pouch cells filled with HD electrolyte was conducted with a C‐rate of 0.05 C, whereas a constant voltage step was incorporated into the charging step at 2.5 V until a current of 1/50 of the nominal capacity was reached. A C‐rate of 0.1 C was applied during the following cycles. Again, a constant voltage step was carried out at the end of charging. Similar to the HD‐filled coin cell, the pouch cells were cycled in a voltage window between 1.5 to 2.5 V vs. Li/Li^+^. The specific energies as well as the energy densities were declared in Table 2. The cell weight of the cell after sealing was used to calculate the gravimetric energy of the discussed pouch cells. The volume for the pouch cells was the product of the average cell thickness and the area of the cathodes. This volume was used to determine the volumetric energy density.

### Surface analysis

The cathode surface morphology was characterized by SEM, and the measurements were carried out on a SU8020 from Hitachi. Images were taken using 5 kV acceleration voltage and a working distance of 4 mm. The pristine binders were analyzed with an acceleration voltage of 2.0 kV to prevent decomposition. The samples were adhered to the aluminum sample holder with conductive carbon tape. The samples were sputtered with gold to enhance surface conductivity, prior to measuring.

The surface morphology during swelling was monitored with a Leica 3D DCM system (Leica Microsystems GmbH). The system was equipped with an objective that has a twenty‐fold magnification. The maximal lateral resolution of the microscope was 0.28 μm and 15 nm in the *z*‐direction. The wavelength of the applied LED was 460 nm. The assembly of the detected images was conducted with Leica Map Premium 6.2.7487. To monitor the swelling behavior of the electrode, a 10 μL droplet of the respective electrolyte was placed cautiously on the surface of the electrode. Subsequently, the dynamic swelling of the electrode was monitored after a certain time interval by conducting measurements. Initially, the height of the cathode coating was measured with the confocal microscope by removing a part of the cathode coating from the aluminum current collector and determining the normalized height of the electrode coating.

## Conflict of interest

The authors declare no conflict of interest.

1

## Supporting information

As a service to our authors and readers, this journal provides supporting information supplied by the authors. Such materials are peer reviewed and may be re‐organized for online delivery, but are not copy‐edited or typeset. Technical support issues arising from supporting information (other than missing files) should be addressed to the authors.

Supporting Information

## Data Availability

Research data are not shared.
