# Supplementary material for: Sustainable Protein‐Based Binder for Lithium‐Sulfur Cathodes Processed by a Solvent‐Free Dry‐Coating Method
Source: ChemSusChem. 2022 Oct 20;15(22):e202201320. doi: 10.1002/cssc.202201320 (PMC9828167; doi:10.1002/cssc.202201320)
Supplement: Supplementary file 1 — Supporting Information [file CSSC-15-0-s001.pdf]

ChemSusChem

Supporting Information

Sustainable Protein-Based Binder for Lithium-Sulfur Cathodes Processed by a Solvent-Free Dry-Coating Method

Florian Schmidt, Sebastian Kirchhoff, Karin Jägle, Ankita De, Sebastian Ehrling, Paul Härtel, Susanne Dörfler,* Thomas Abendroth, Benjamin Schumm, Holger Althues, and Stefan Kaskel© 2022 The Authors. ChemSusChem published by Wiley-VCH GmbH. This is an open access article under the terms of the Creative Commons Attribution License, which permits use, distribution and reproduction in any medium, provided the original work is properly cited.

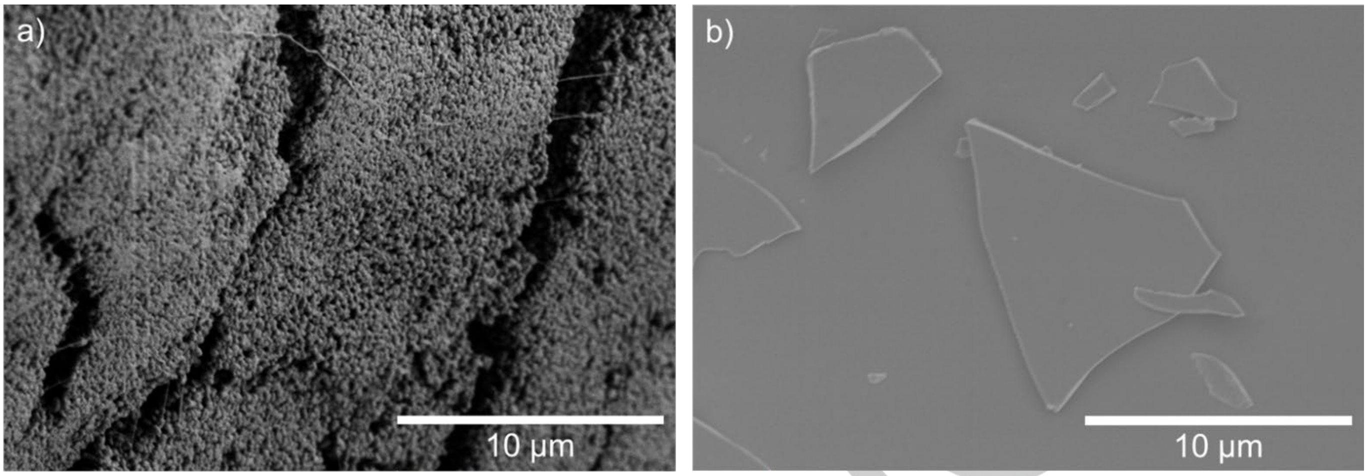

FigureS 1: SEM micrographs of pristine a) PTFE and b) sericin. The measurements were conducted with an acceleration voltage of 2.0 kV.

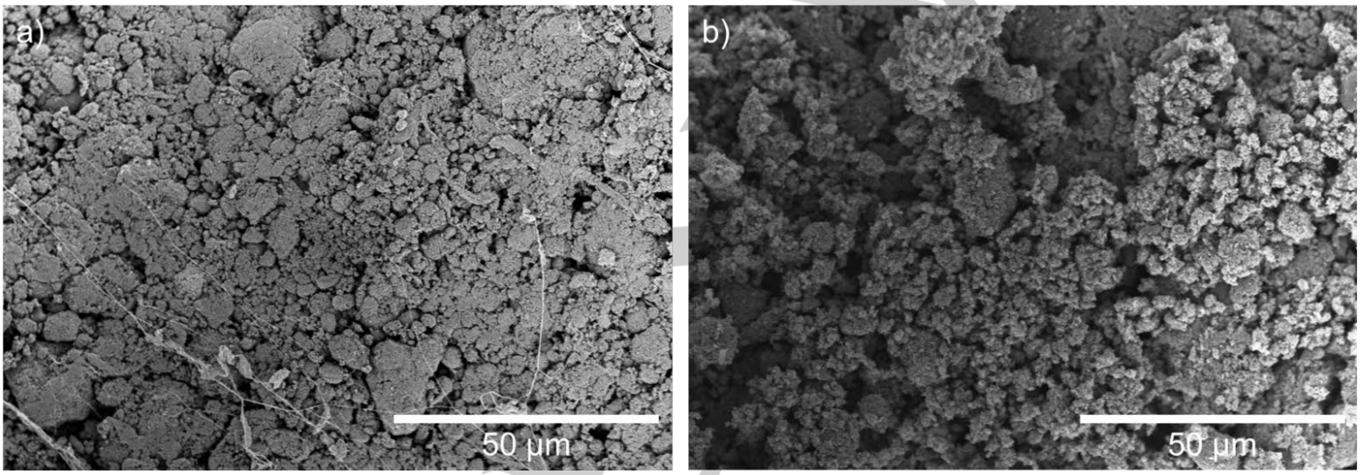

FigureS 2: SEM micrographs of a) PTFE- and b) sericin-based dry-film cathodes. The micrographs were taken with an acceleration voltage of 2.0 kV.

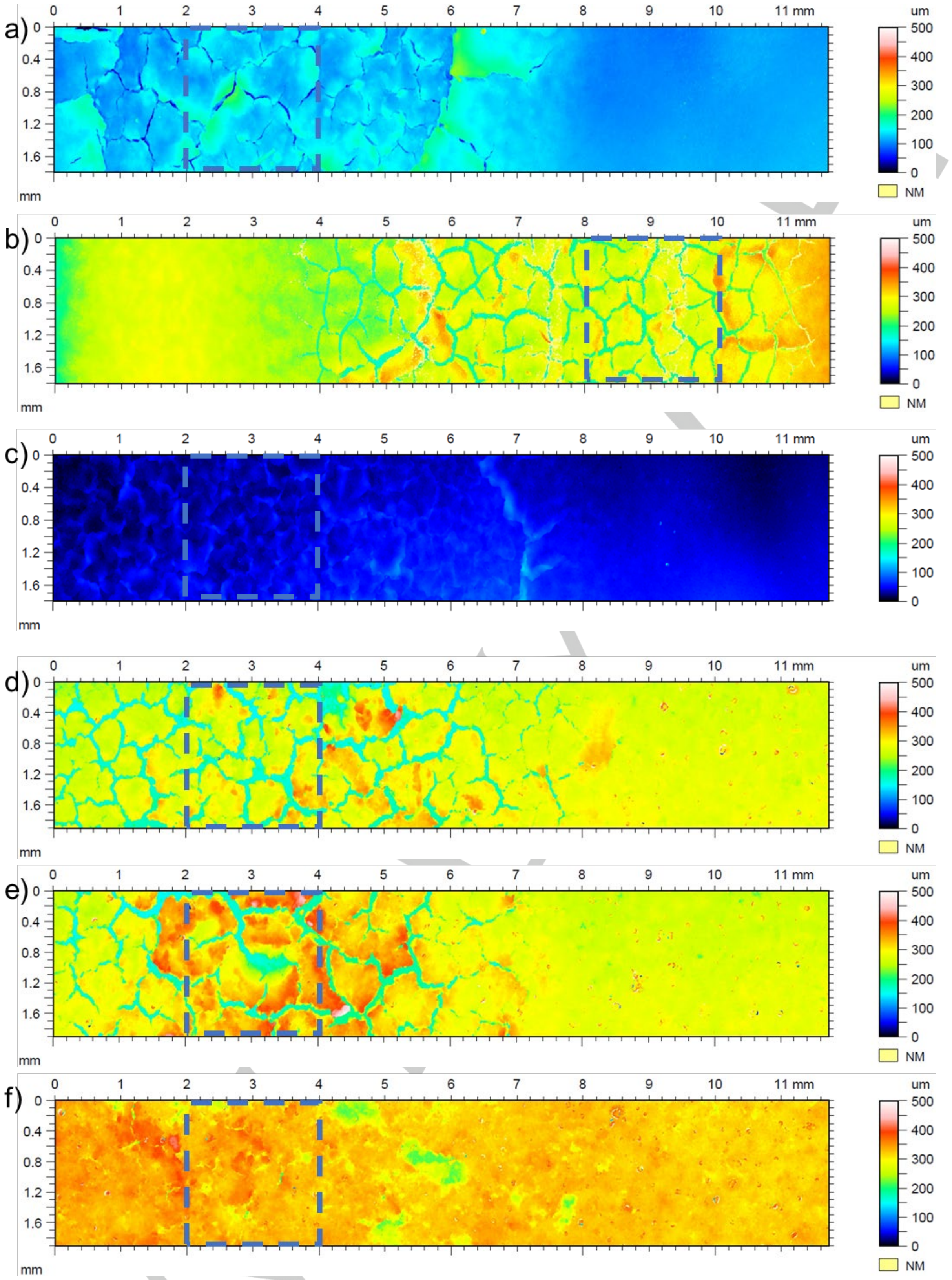

FigureS 3: Confocal microscopy of PTFE-based (a-c) and sericin-based (d-f) cathodes after contact with 10 µL DD (a,d), HD (b,e) or TT (c,f) electrolyte. The cathode area where the electrolyte was deposited is highlighted. On the right hand side, the surface that was not wetted/affected by the electrolyte is shown as well.

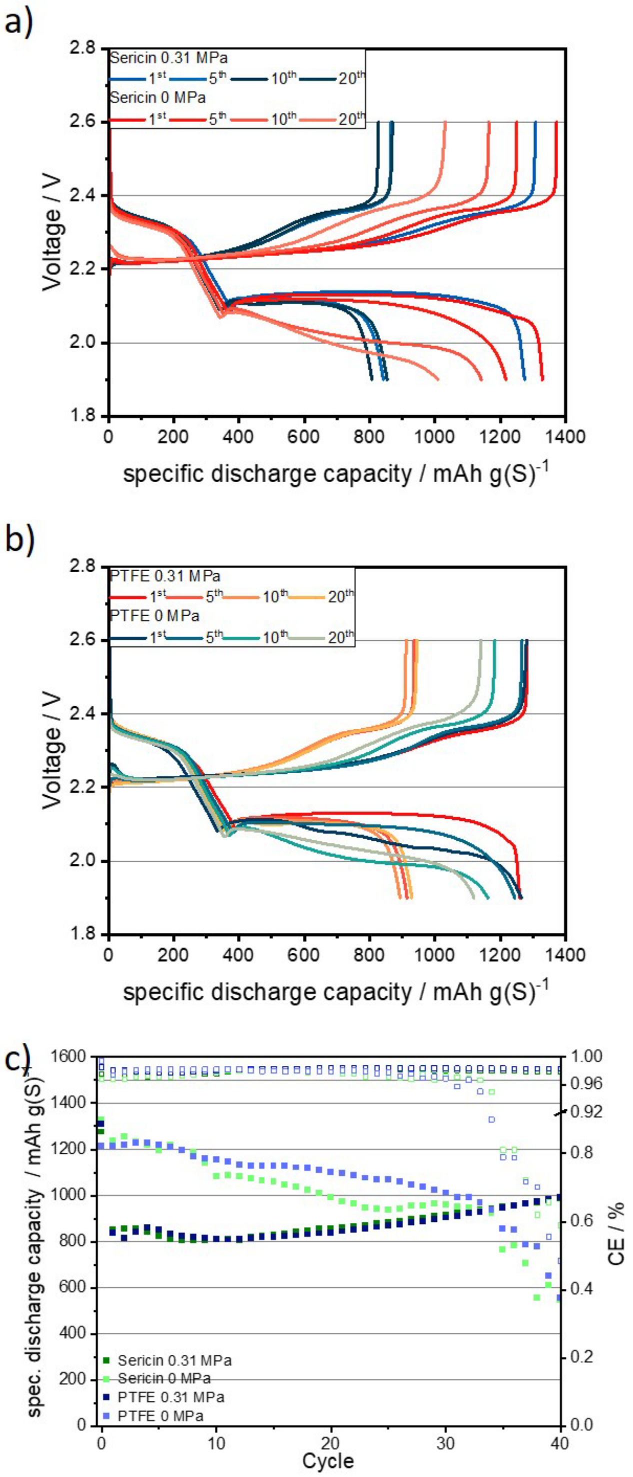

FigureS 4: Voltage profiles of DD-filled five layer pouch cells with a) sericin- and b) PTFE-based cathodes. c) Galvanostatic cycling of sericin- and PTFE-based cathodes in five-layered pouch cells. The cells were tested with an external uniaxial pressure of 0.31 MPa or without pressure application. An E/S ration of 4.5 µL mg(S)$^{-1}$ was applied. The same testing procedure as for the coin cells was conducted
